# *parsomics*: a data-driven framework for metagenomics data integration powered by a local relational database

**DOI:** 10.1093/bioadv/vbag049

**Published:** 2026-02-15

**Authors:** Pedro Sader de Azevedo, Meiski Mariá Vedovatto, Pedro Coelho Gimenes de Freitas, Rafaela Beatriz Silva Luz, Rodrigo Silva Araujo Streit, Gabriela Felix Persinoti

**Affiliations:** Brazilian Biorenewables National Laboratory (LNBR), Brazilian Center for Research in Energy and Materials (CNPEM), Campinas, Sao Paulo, 13083-100, Brazil; Department of Computer Engineering, State University of Campinas (UNICAMP), Campinas, Sao Paulo, 13083-970, Brazil; Brazilian Biorenewables National Laboratory (LNBR), Brazilian Center for Research in Energy and Materials (CNPEM), Campinas, Sao Paulo, 13083-100, Brazil; Ilum School of Science, Brazilian Center for Research in Energy and Materials (CNPEM), Campinas, Sao Paulo, 13083-100, Brazil; Ilum School of Science, Brazilian Center for Research in Energy and Materials (CNPEM), Campinas, Sao Paulo, 13083-100, Brazil; Brazilian Biorenewables National Laboratory (LNBR), Brazilian Center for Research in Energy and Materials (CNPEM), Campinas, Sao Paulo, 13083-100, Brazil; Brazilian Biorenewables National Laboratory (LNBR), Brazilian Center for Research in Energy and Materials (CNPEM), Campinas, Sao Paulo, 13083-100, Brazil

## Abstract

**Motivation:**

Metagenomics enables the analysis of complex microbial communities directly from environmental samples, resulting in massive datasets that are processed using multiple tools and workflows. Data integration is key for metagenomics research, however, challenges in data organization and management locally remain open in existing workflows.

**Results:**

We present *parsomics*, a lightweight and extensible data management tool designed for efficient local storage, organization, and integration of metagenomic analysis results. Built upon PostgreSQL and implemented in Python, *parsomics* leverages a user-defined configuration file to automatically construct a relational database tailored to metagenomics-based data. It is user-friendly, easy to deploy, and implements modular plugin-based extensions to support diverse data types and outputs. *parsomics* can be installed in every major GNU/Linux environment and currently focuses on prokaryotic metagenomics analysis.

**Availability and implementation:**

*parsomics* is an open-source project and its source code is available at https://gitlab.com/parsomics under the GPLv3 license. Comprehensive documentation can be found at https://parsomics.org and https://api.parsomics.org.

## 1 Introduction

Metagenomics is an effective approach for analyzing and characterizing microbial communities and is particularly suitable for exploring the vast diversity of uncultured microorganisms. Advances in high-throughput and cost-effective sequencing technologies have pushed the discovery and availability of such microbes as metagenome-assembled genomes (MAGs), which represent an unprecedented resource for understanding microbial diversity, ecology, and function (Haft *et al*. 2024). However, the scale and complexity of metagenomic datasets represents a challenge for data processing and management, especially for small and mid-sized research groups with limited computational infrastructure (Markowitz *et al*. 2015). As the volume of raw sequences and associated metadata increases, the need for efficient strategies to store, organize and visualize large datasets becomes critical (O’Donoghue 2021, Aplakidou *et al*. 2024).

In addition to the volume, metagenomic datasets subjected to standard analysis workflows typically involve multiple steps, including *de novo* assembly, binning, dereplication, taxonomic assignment, gene prediction, and functional annotation. Each of these steps relies on distinct bioinformatic tools that generate several outputs in varied formats (Yang *et al*. 2021, Zhou *et al.* 2022). This level of complexity, specifically the fragmented information distributed across multiple intermediate files, highlights the need for a systematic and flexible solution to integrate outputs across different tools, workflows and different research projects.

Several platforms have been developed to support and manage metagenomic data analysis, each with distinct goals. For instance, MG-RAST (Meyer *et al*. 2008), Qiita (Gonzalez *et al*. 2018), and MGnify (Abueg *et al*. 2024) are web-based data analysis portals for executing bioinformatics workflows with a graphical user interface. Importantly, these portals emphasize metadata integration in standardized pipelines. KBASE (Arkin *et al*. 2018), and NFDI4Microbiome (NFDI4Microbiota n.d.), in turn, focus on data integration and community-based sharing while allowing minor pipeline customization. These web applications provide non-specialists with accessible bioinformatic tools designed to ensure scalability, reproducibility, and interoperability.

Although these cloud-based platforms contribute to the democratization of complex bioinformatic analyses, they may present some limitations. Given the fast-evolving landscape of metagenomic-related algorithms (Zhang *et al*. 2023, Han *et al.* 2025) online platforms developers may not adapt rapidly enough to integrate the latest methodological advances. Hence, relying on third-party platforms that lie beyond the user’s control may hinder the adoption of state-of-the-art tools. In addition, depending on the available network infrastructure, users may face significant latency when uploading terabyte-scale raw data to a web-based system. Moreover, private organizations may also face usage challenges concerning security and legal permissions, as many publicly available tools restrict commercial and profit-oriented use.

Locally hosted solutions for storing and managing metagenomic data address these issues; however, currently available tools still lack important features. For example, MicrobeDB (Langille *et al*. 2012) was designed to download and build a SQL database of microbial genomes, but it is limited to retrieving publicly available sequences. MyPhyloDB (Manter *et al.* 2016), although described as a metagenomic data manager, is primarily for metabarcoding data. Alternatively, MetaHCR (Marks *et al.* 2018) represents a more comprehensive framework for storing, managing and searching metagenomic data and associated metadata. However, its reliance on the IMG platform for annotation limits interoperability with other tools. To date, metaXplor (Sempéré *et al*. 2021) is the most extensive system for metagenomic data integration and management. Nevertheless, it lacks support for programmatic querying, limiting its integration into scripted analysis.

Here, we present *parsomics*, a local data management tool designed to efficiently organize high throughput-derived data generated from prokaryotic metagenomics workflows. *parsomics* supports the automatic parsing and integration of diverse file formats, produced throughout typical metagenomics pipelines, into the *parsomics* Local Relational Database (pLRDB), a standardized, cohesive, queryable representation of data and associated metadata. The pLRDB can be accessed and extended using widely adopted open standards, ensuring that *parsomics* remains accessible, extensible and compatible with existing and upcoming tools. This makes it a flexible and powerful framework for local metagenomics data management.

## 2 Methods

### 2.1 Implementation

The *parsomics* project is composed of multiple modules written in Python and distributed through the Python Package Index (PyPI), for convenient installation. This modular architecture improves the Separation of Concerns (SoC) (Mitchell R (Ed.)) and facilitates the creation of reusable software components. The *parsomics* core modules are listed below:

- *parsomics-core*: library for parsing files and populating the pLRDB.
- *parsomics-api-server*: REST API server to interact with the pLRDB.
- *parsomics-cli*: Command-line interface (CLI) for managing and running *parsomics*.

As its name suggests, the *parsomics-core* module contains the core logic of the project. It is a Python library responsible for connecting to a local PostgreSQL database server that hosts the *parsomics* Local Relational Database (pLRDB). This module defines the pLRDB schema, creates its tables, indexes, and constraints, and populates the database with data specified in a user-defined configuration file. To ensure high performance, the data insertion routines in *parsomics-core* have been extensively optimized using memorization (Norvig 1991) and batch operations to reduce disk access and minimize I/O wait time (Supplementary Fig. S1). As a library, *parsomics-core* does not operate independently; its functionality is invoked by the *parsomics-cli* module, which serves as the primary command-line interface for user interaction.

**Figure 1:**
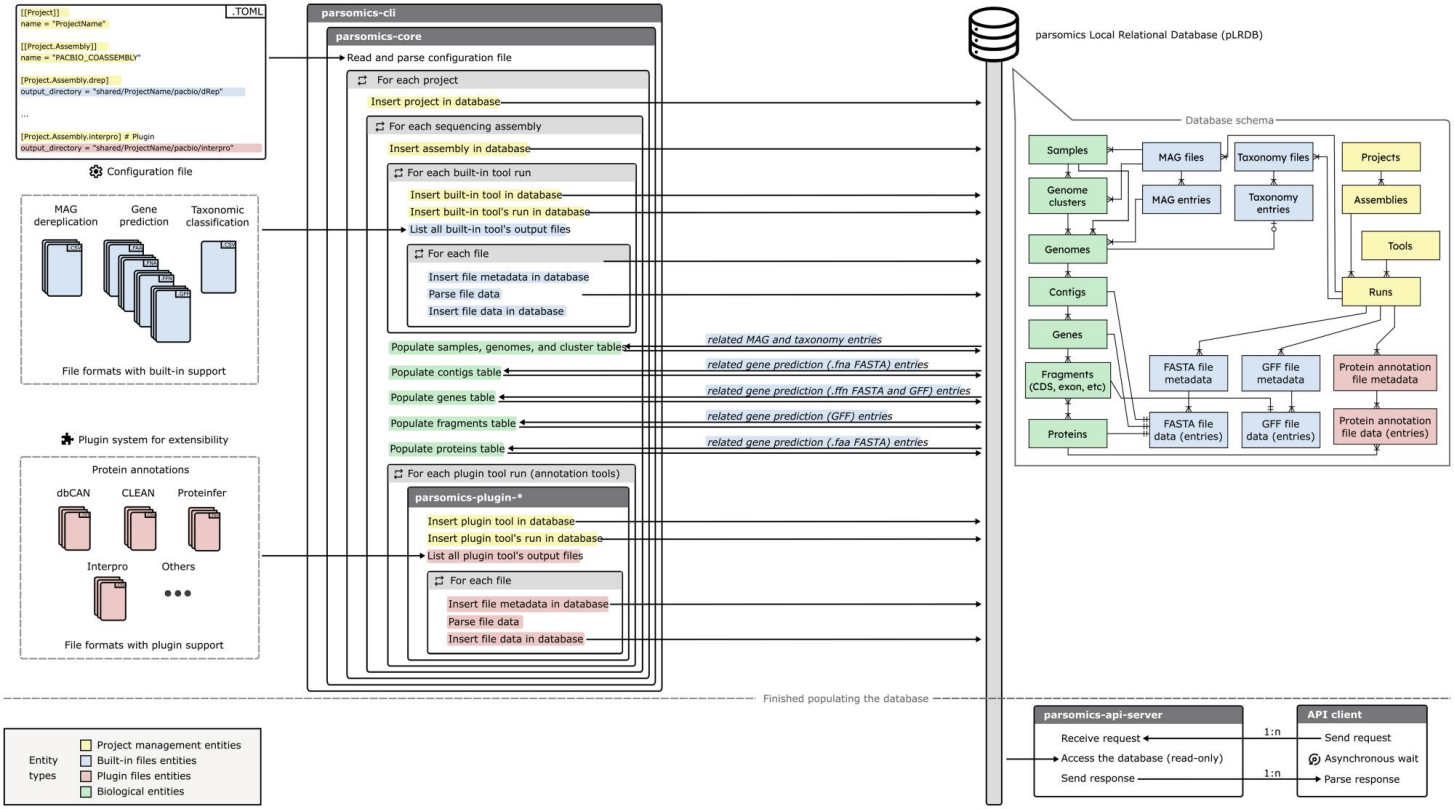
*parsomics* general execution flow. The process begins with a user-defined configuration file, which specifies the data to be processed. The *parsomics-cli* module uses functions defined in the *parsomics-core* library to read the configuration file and orchestrate the insertion of data into the pLRDB. Since biological entities are associative entities, they are assembled using foreign keys obtained from earlier stages of insertion (indicated in italic). The simplified version of pLRDB schema is shown on the right, color-coded with its categories. Finally, the *parsomics-api-server* exposes a REST API for external clients to get read-only access to the pLRDB.

The pLRDB schema comprises 28 tables, organized into three categories: Project Management Entities, File Entities, and Biological Entities. The first category stores metadata about workflow execution, including tools used, sources, versions, and timestamps. File Entities store data parsed from standard output file formats such as FASTA, GFF, and TSV. Biological Entities represent abstractions such as genomes, contigs, genes, and proteins. These tables integrate and contextualize data across multiple files, allowing queries that reflect biological relationships rather than file-specific representations. This schema establishes the foundation for semantically rich, biologically meaningful analyses. A simplified Entity-Relationship (ER) diagram of the schema is at the top right corner of Fig. 1, while the full ER diagram is provided in Supplementary Fig. S2.

*parsomics* is designed to integrate and store information from metagenome-assembled genomes. Any tool or pipeline for *de novo* assembly and binning producing standard FASTA and GFF files are compatible without modification. This includes MAG-recovery tools and pipelines such as MetaWRAP (Uritskiy *et al.* 2018), MaxBin2 (Wu *et al.* 2016), MetaBAT2 (Kang *et al.* 2019), and COMEbin (Wang *et al*. 2024), among others. For MAGs dereplication *parsomics* is compatible with dRep (Olm *et al*. 2017) and gene annotation tools such as Prokka (Seemann 2014). *parsomics* is also compatible with the widely used taxonomic classifier GTDB-Tk (Chaumeil *et al*. 2019). The benchmarking section illustrates this compatibility through end-to-end processing of datasets that were *de novo* assembled, binned, dereplicated, annotated, and classified using deployed tools with no additional preprocessing required. *parsomics* pLRDB is designed in accordance with the first, second, and third normal forms of Relational Database Theory (Codd 1972), promoting data consistency and eliminating redundancies by enforcing a Single Source of Truth (Queiroz *et al.* 2024) for each data point. This level of normalization is especially beneficial in the metagenomics domain, where datasets are inherently large, diverse, and highly interconnected. It supports precise modeling of relationships between biological and computational entities, while enabling flexible and composable querying across the database. Considerable effort was also invested in enforcing data integrity. Every database object is verified using the Pydantic validation library, ensuring that only well-formed and acceptable data is mapped and committed to the pLRDB. Additionally, multiple uniqueness constraints safeguard the database against redundancy.

While normalization and validation impose strict rules for the data that goes into the pLRDB, the system presents a high level of flexibility through the *parsomics* plugin architecture. This system is used to implement parsers for protein annotation and was designed to accommodate the ever-expanding landscape of bioinformatics tools, which are frequently improved or replaced by newer alternatives. By decoupling tool-specific logic from the core of the codebase, the plugin system enables sustainable extensibility without compromising the project’s maintainability and stability.

Plugins in *parsomics* work by providing functions that parse the output of a specific annotation tool into a unified format, populating the protein annotations tables within the pLRDB. Plugins are constrained to populate existing tables and do not modify the underlying database schema, which is important for ensuring consistency and long-term interoperability. Users can list, install, and uninstall plugins through the “*parsomics plugin*” command provided by the *parsomics-cli*. The current set of available plugins is:

- *parsomics-plugin-interpro*: adds support for protein annotations generated using InterProScan (Blum *et al*. 2025).
- *parsomics-plugin-dbcan*: adds support for carbohydrate active enzymes annotations generated using dbCAN tool (Zheng *et al*. 2023).
- *parsomics-plugin-clean*: enables integration of protein annotations from the machine learning-based tool CLEAN (Yu *et al.* 2023).
- *parsomics-plugin-proteinfer*: supports annotations from the ML-based tool ProteInfer (Sanderson *et al*. 2023).

Like modules, plugins are implemented as standard Python packages and distributed through the Python Package Index (PyPI), eliminating the need for custom plugin specifications. Creating a new plugin is straightforward using the official template and accompanying tutorial, allowing researchers to rapidly integrate emerging tools while maintaining compatibility with the core data model. For use cases beyond the scope of the plugin framework, *parsomics* can be further extended directly through its open-source codebase. This dual strategy, including plugin extensibility and open-source access, aims to foster a growing ecosystem of community-driven contributions and extensions for *parsomics*.

Once the pLRDB is populated with metagenomics data, users can interact with it programmatically. Programmatic access is provided by direct SQL queries or via language‐native object‐relational mappers (e.g., SQLAlchemy) and is further simplified by the *parsomics‐api‐server* module, which exposes a REST API for data retrieval and manipulation. This API enables the development of custom applications for data visualization, data mining, and data analysis. As an example, a graphical user interface for data mining and visualization named *parsomics-explorer* is currently under active development (Supplementary Fig. S3). It aims to further lower the barrier to data exploration by allowing researchers to query, browse, and interpret metagenomic data without requiring prior experience with SQL or programming, complementing the programmatic workflows already supported by the platform. The complete execution flow of *parsomics*, including all its modules, can be seen in Fig. 1.

Together, the architectural implementation, including the modular design, schema normalization, object validation, plugin system, open-source codebase, and REST API, enforce best practices in scientific data management. In particular, they promote the adoption of FAIR principles (Wilkinson *et al.* 2016) in metagenomics data, by supporting Findability through structured queries, Accessibility via multiple interfaces, Interoperability through common standards, and Reusability by encouraging community-driven extensions. This commitment is further emphasized by comprehensive user and developer documentation, including detailed specifications of the pLRDB schema, covering the attributes, constraints, and indexes of each table, as well as, multiple examples of queries using SQL, Python with ORM library, and the REST API.

### 2.2 Installation and usage

*parsomics* is published as a metapackage in PyPI, which encompasses all core modules and plugins. This metapackage simplifies installation and ensures version compatibility across components. It can be installed using any standard Python package manager, such as pip or conda, facilitating easy deployment in diverse environments.

After installation, the *parsomics* setup command guides the user through the configuration process to initialize the system and populate the pLRDB. Once the database is populated, it can be accessed through multiple supported interfaces, including direct SQL access, Object-Relational Mapping (ORM) libraries, or the built-in REST API, as detailed in the Implementation section. Query examples are provided in Supplementary Material Figs. S4 and S5; additional examples and use cases are available in the project documentation.

The *parsomics* command-line interface also includes utilities for managing plugins, such as listing, installing, uninstalling, as well as, handling configuration files including viewing, editing, and locating them. For detailed usage information, a —help flag can be appended to any command and subcommand to provide further usage details and options.

### 2.3 Benchmark datasets

Benchmark datasets include both publicly available datasets (NCBI Accession PRJNA110382; Santos *et al*. 2025) and the Critical Assessment of Metagenomes Interpretation (CAMI I high complexity; Sczyrba *et al.* 2017), as well as, other internal projects representing typical metagenomic research scenarios (Supplementary Table S3). Each dataset was subjected to *de novo* assembly using MEGAHIT (Li *et al*. 2015), and metagenome-assembled genomes were recovered using the MetaWRAP pipeline (Uritskiy *et al*. 2018) with default settings. High-quality and non-redundant genomes were assessed using CheckM2 (Chklovski *et al*. 2023) and dRep (Olm *et al*. 2017), respectively. Unique MAGs were then submitted to gene annotation using Prokka (Seemann 2014) and taxonomy assignment using GTDB-Tk (Chaumeil *et al*. 2019). For the CAMI I High-Complexity dataset, pre-computed assemblies provided for the challenge were used to recover MAGs using MaxBin2 (Wu *et al*. 2016), MetaBAT2 (Kang *et al.* 2019), and COMEbin (Wang *et al.* 2024), whereas downstream analyses followed the same workflow applied to the other datasets.

## 3 Results and discussion

To evaluate the performance and scalability of *parsomics*, we benchmarked its execution on six metagenomic projects of varying sizes. These projects included both publicly available datasets and internal projects, representing typical use cases in metagenomics research.

Processing times scaled with dataset size and annotation complexity. For example, one project comprising 111 MAGs recovered from soil covered with sugarcane bagasse with two associated protein annotation sources per MAG (∼800 MB total), was processed in approximately 23 minutes. A large-scale dataset with 1215 MAGs associated with manatee gut microbiota with three protein annotations sources per MAG (∼12.1 GB total), required 5.11 hours for processing. Across all benchmark cases, the resulting pLRDB occupied approximately the same disk space as the original data or less, due to PostgreSQL’s native compression features, indicating consistent performance and efficient storage.

All benchmarks were run on a standard laptop equipped with an Intel Core i5 vPro processor, 40 GB of RAM, and a 1 TB SSD, demonstrating that *parsomics* operates on consumer-grade hardware. Like most database-centric applications, *parsomics* is I/O bound, with the majority of execution time spent on disk read/write operations (Supplementary Fig. S6). Importantly, this processing step is only performed once to populate the pLRDB; upon completion, data becomes permanently available and can be queried efficiently, which typically execute within milliseconds.

In contrast to existing web services, *parsomics* runs entirely on-premise. This local-first approach reduces dependency on third-parties services and mitigates concerns regarding data confidentiality and regulatory compliance. Furthermore, the system remains lightweight and portable, requiring no high-performance computing (HPC) infrastructure. This allows researchers to reserve HPC resources for computationally intensive tasks such as metagenomic *de novo* assembly and annotation, while managing and querying data can be done efficiently on standard laboratory hardware.

Furthermore, *parsomics* enables exploratory intra and inter project queries that are difficult to perform when data are stored in flat files. To illustrate its utility for enzyme discovery representative query examples are provided in Supplementary Figs. S4 and S5 with corresponding results shown in Supplementary Tables S1 and S2.

Carbohydrate-active enzymes (CAZymes) play central roles in biomass depolymerization and present diverse biotechnological applications. Using *parsomics*, we integrated CAZyme annotations from multiple projects and executed a single SQL query to retrieve all CAZymes restricted to taxa classified as taxonomic novelties, according to GTDB-Tk (Chaumeil *et al.* 2019). This query returned 8252 enzymes from different metagenomic projects and it was executed in 10 s (Supplementary Fig. S4, Supplementary Table S1), enabling a comprehensive overview of the CAZymes functional repertoire encoded by novel taxa. To narrow down this sequence universe, a more targeted query focusing on enzymes from glycoside hydrolase family GH5, a CAZyme group with broad activity related to biomass breakdown was executed. This query explored multiple projects stored in the pLRDB and returned, for each metagenome-assembled genome (MAG) classified as a taxonomic novelty, the number of proteins annotated as GH5. In total, 97 MAGs were identified across independent metagenomic projects in 1.35 s, enabling rapid prioritization of candidates MAGs for in-deep investigation. These queries can be automated via the REST API to support iterative searches across additional enzyme families. These examples illustrate how *parsomics* supports exploratory analyses and hypothesis-driven queries through a unified, automated, and reproducible framework.

While the current implementation of *parsomics* already provides support for standardized inputs, such as FASTA and GFF files, opportunities remain for expanding interoperability in future versions. In particular, upcoming development efforts will focus on broadening support for heterogeneous output formats generated by a wider range of metagenomic tools, to minimize the need for manual reformatting, and further accelerate the integration across diverse workflows.

In summary, *parsomics* offers a robust and extensible solution for organizing and managing metagenomic data locally. Its architecture provides a practical balance between standardization and flexibility, while also promoting openness and reusability through its plugin system and open-source philosophy. It is a system built to evolve with the rapidly changing landscape of bioinformatics, and to empower researchers to extract more value from their data with confidence and control.

## Supplementary Material

vbag049_Supplementary_Data

## Data Availability

Source code and reference test dataset are available at https://gitlab.com/parsomics and https://zenodo.org/records/15720967, respectively. *parsomics* documentation is provided at https://parsomics.org and https://api.parsomics.org.
