# Supplementary material for: parsomics: a data-driven framework for metagenomics data integration powered by a local relational database
Source: Bioinform Adv. 2026 Feb 15;6(1):vbag049. doi: 10.1093/bioadv/vbag049 (PMC13006488; doi:10.1093/bioadv/vbag049)
Supplement: vbag049_Supplementary_Data [file vbag049_supplementary_data.pdf]

Supplementary Materials

parsomics: a data-driven framework for metagenomics data integration powered by a local relational database

Pedro Sader de Azevedo[1,2], Meiski Mariá Vedovatto[2], Pedro Coelho Gimenes de Freitas[3], Rafaela Beatriz Silva Luz[3], Rodrigo Silva Araujo Streit[2], Gabriela Felix Persinoti[2,*]

[1] Computer Engineering Department, State University of Campinas (UNICAMP), Campinas, Brazil.
[2] Brazilian Biorenewables National Laboratory (LNBR), Brazilian Center for Research in Energy and Materials (CNPEM), Campinas, Brazil.
[3] Ilum School of Science, Brazilian Center for Research in Energy and Materials (CNPEM), Campinas, Brazil.

*Corresponding author. Brazilian Biorenewables National Laboratory (LNBR), Brazilian Center for Research in Energy and Materials (CNPEM), R. Giuseppe Máximo Scolfaro, 10000 - Bosque das Palmeiras, Campinas, São Paulo, Brazil. E-mail: gabriela.persinoti@lnbr.cnpem.br

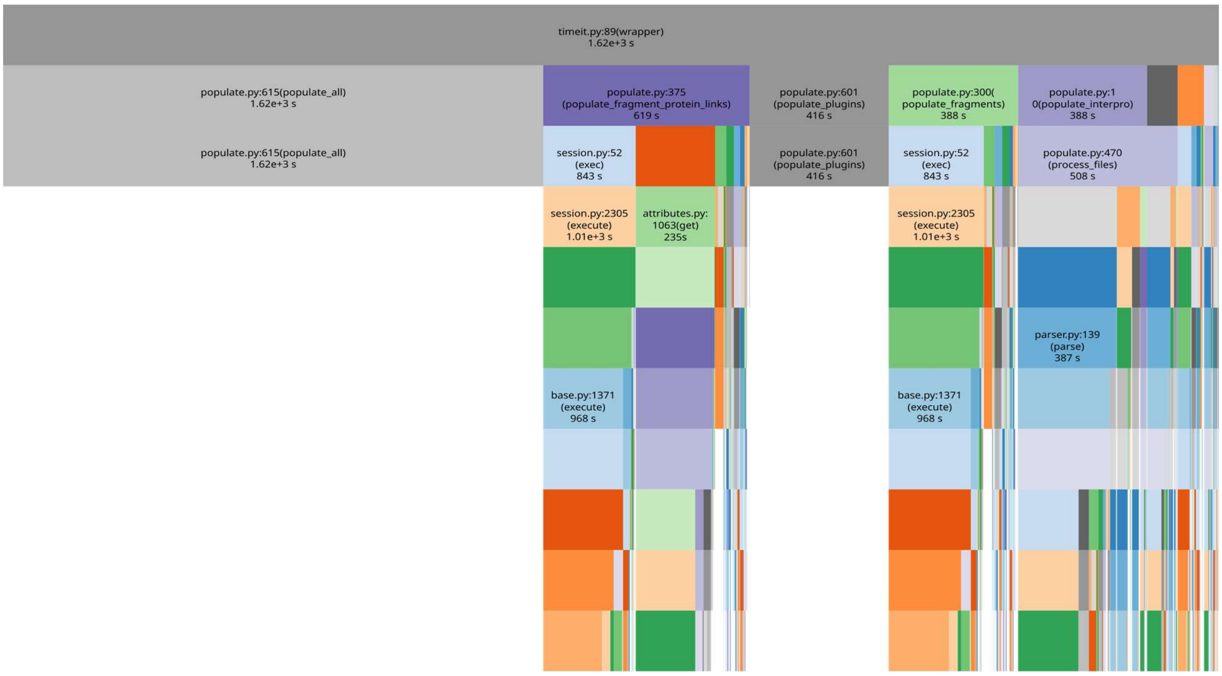

Figure S1: Top-down flamegraph of *parsomics* benchmark results. The profile was generated using a metagenomic project containing 110 MAGs. This visualization shows the execution time for each level of *parsomics-core* call stack, helping to identify performance bottlenecks at different stages of processing. In particular, it highlights which pLRDB tables are the most time-consuming to populate. Insights from this profiling guided several optimization efforts. This benchmarking profile was generated using cProfile (Roskind, Mullender and Rossum 2024) and visualized with SnakeViz (Davis 2024).

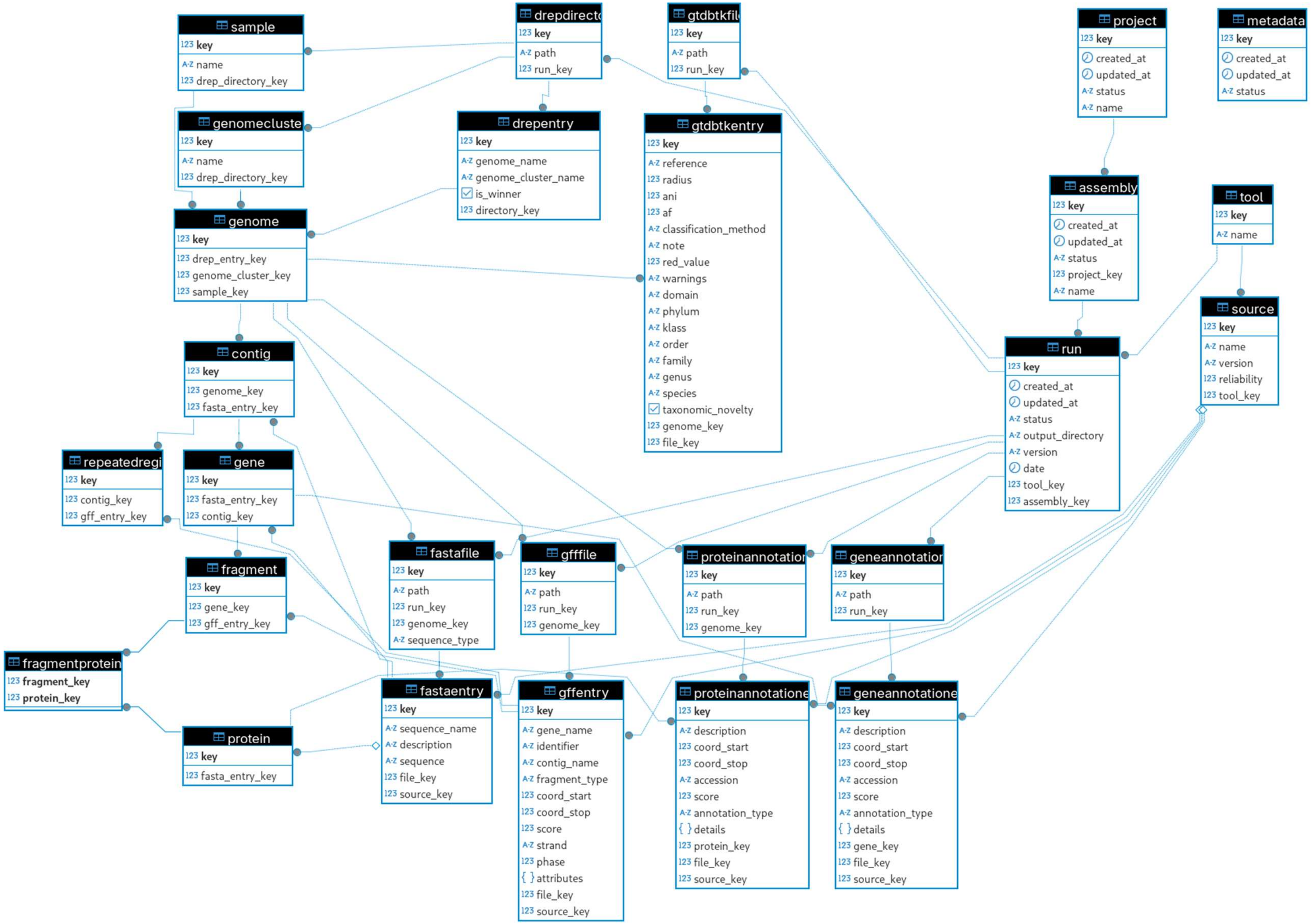

Figure S2: Entity-Relationship (ER) diagram of *parsomics* Local Relational Database schema (pLRDB).

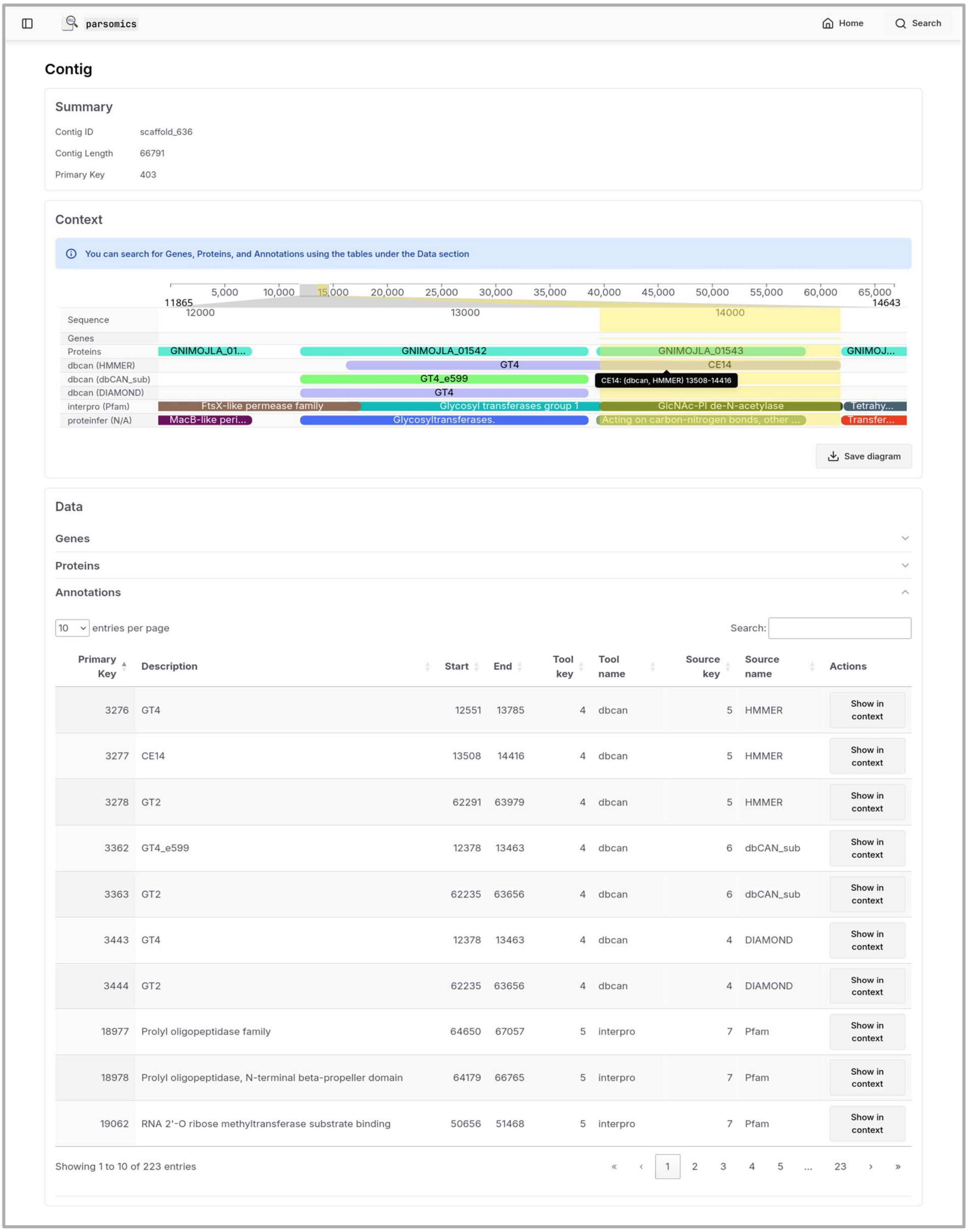

Figure S3: Example of *parsomics-explorer*, a graphical user interface for data mining and data visualization that interacts with the pLRDB using the REST API.

```sql
SELECT
    fastaentry.sequence_name AS seq_name,
    proteinannotationentry.description,
    source.name AS source_name,
    drepentry.genome_name AS genome_name,
    sample.name AS sample_name,
    gtdbtkentry.taxonomic_novelty AS taxonomic_novelty,
    assembly.name AS assembly_name
FROM proteinannotationentry
JOIN protein ON proteinannotationentry.protein_key = protein.key
JOIN fragmentproteinlink ON protein.key = fragmentproteinlink.protein_key
JOIN fragment ON fragmentproteinlink.fragment_key = fragment.key
JOIN gene ON fragment.gene_key = gene.key
JOIN contig ON gene.contig_key = contig.key
JOIN genome ON contig.genome_key = genome.key
JOIN sample ON genome.sample_key = sample.key
JOIN gtdbtkentry ON gtdbtkentry.genome_key = genome.key
JOIN drepentry ON genome.drep_entry_key = drepentry.key
JOIN gffentry ON fragment.gff_entry_key = gffentry.key
JOIN fastaentry ON fastaentry.key = protein.fasta_entry_key
LEFT JOIN source ON proteinannotationentry.source_key = source.key
JOIN proteinannotationfile ON proteinannotationentry.file_key =
proteinannotationfile.key
JOIN run ON proteinannotationfile.run_key = run.key
JOIN assembly ON run.assembly_key = assembly.key
JOIN tool ON run.tool_key = tool.key
WHERE taxonomic_novelty = true AND
    fragment_type = 'CDS' AND
    tool.name = 'dbcan' AND
    source.name = 'HMMER' AND
    taxonomic_novelty;
```

Figure S4: Example of a query written in SQL, which traverses multiple tables in pLRDB to return CAZyme protein annotations from metagenome-assembled genomes (MAGs) that were annotated as taxonomic novelties.

Table S1: Preview of first 15 out of 8.252 CAZymes identified in different metagenomic projects. These results correspond to the query shown in Figure S4, which was executed in 10 seconds.

seq_name	description	source_name	genome_name	sample_name	Taxonomic novelty	assembly_name
HAFJLCCH_00197	CE1	HMMER	PLS.PVC-P2_S42.bin.9	PLS.PVC-P2_S42	TRUE	ILLUMINA-PLASTICS
HAFJLCCH_00423	GH29	HMMER	PLS.PVC-P2_S42.bin.9	PLS.PVC-P2_S42	TRUE	ILLUMINA-PLASTICS
DLEEADGK_00326	GH18	HMMER	PLS.PP-P10_S41.bin.15	PLS.PP-P10_S41	TRUE	ILLUMINA-PLASTICS
DLEEADGK_00563	GT4	HMMER	PLS.PP-P10_S41.bin.15	PLS.PP-P10_S41	TRUE	ILLUMINA-PLASTICS
APPAMAKP_00269	GH23	HMMER	PLS.LDPE-P6_S17.bin.2	PLS.LDPE-P6_S17	TRUE	ILLUMINA-PLASTICS
GOEIOMOB_00044	GT2	HMMER	PBM.casm.bin.44	PBM.casm	TRUE	ILLUMINA-PBM
GOEIOMOB_00771	CE4	HMMER	PBM.casm.bin.44	PBM.casm	TRUE	ILLUMINA-PBM
GOEIOMOB_00782	GH39	HMMER	PBM.casm.bin.44	PBM.casm	TRUE	ILLUMINA-PBM
GOEIOMOB_00905	GH10	HMMER	PBM.casm.bin.44	PBM.casm	TRUE	ILLUMINA-PBM
GOEIOMOB_00979	GH39	HMMER	PBM.casm.bin.44	PBM.casm	TRUE	ILLUMINA-PBM
KJJFLJFK_000081	GH3	HMMER	L1.bin.96	L1	TRUE	ILLUMINA
JCFGABNC_000373	AA3	HMMER	L1.bin.71	L1	TRUE	ILLUMINA
JCFGABNC_00866	GT4	HMMER	L1.bin.71	L1	TRUE	ILLUMINA
JCFGABNC_00245	GH109	HMMER	L1.bin.71	L1	TRUE	ILLUMINA
EAGNCIBC_00526	AA6	HMMER	L1.bin.8	L1	TRUE	ILLUMINA

```sql
SELECT
    DISTINCT
project.name AS Project_name,
gtdbtkentry.domain AS "Domain",
gtdbtkentry.phylum AS "Phylum",
gtdbtkentry.klass AS "Class",
gtdbtkentry.order AS "Order",
gtdbtkentry.family AS "Family",
gtdbtkentry.genus AS "Genus",
    COUNT(drepentry.genome_name) AS "GH5 per MAG",
drepentry.genome_name AS "MAG_name"
FROM project
JOIN assembly ON assembly.project_key = project.key
JOIN run ON assembly.key = run.assembly_key
JOIN proteinannotationfile ON run.key = proteinannotationfile.run_key
JOIN proteinannotationentry ON proteinannotationfile.key =
proteinannotationentry.file_key
JOIN genome ON proteinannotationfile.genome_key = genome.key
JOIN drepentry ON genome.drep_entry_key = drepentry.key
JOIN gtdbtkentry ON genome.key = gtdbtkentry.genome_key
WHERE
    gtdbtkentry.taxonomic_novelty AND
    proteinannotationentry.description ~* '\yGH5(_\w+)?\y'
GROUP BY
    project.name,
    gtdbtkentry.domain,
    gtdbtkentry.phylum,
    gtdbtkentry.klass,
    gtdbtkentry.order,
    gtdbtkentry.family,
    gtdbtkentry.genus,
    gtdbtkentry.species,
    drepentry.genome_name;
```

Figure S5: Query written in SQL to explore multiple projects in pLRDB and return the number of proteins annotated as Glycoside Hydrolase family GH5 from each MAG identified as taxonomic novelty.

Table S2: Preview of 15 out of 97 fetched lines from three metagenomic projects of varying sizes, corresponding to the query shown in Figure S5, executed in 1.37 seconds.

project_name	Domain	Phylum	Class	Order	Family	Genus	GH5 per MAG	MAG_name
antillean-manatee	Bacteria	Actinomycetota	Coriobacteriia	Coriobacteriales	Eggerthellaceae	UMGS1502	4	PBM.Pintada.bin.27
antillean-manatee	Bacteria	Actinomycetota	Coriobacteriia	Coriobacteriales	QAMH01	CACZQA01	1	PBM.casm.bin.435
antillean-manatee	Bacteria	Bacillota	Bacilli	Acholeplasmatales	Anaeroplasmataceae	CAKQDS01	6	PBM.Pintada.bin.26
antillean-manatee	Bacteria	Bacillota	Bacilli	RFN20	CAG-449		4	PBM.Pintada.bin.11
antillean-manatee	Bacteria	Bacillota_A	Clostridia	Christensenellales			4	PBM.Mirim.bin.17
bagasse-all	Bacteria	Acidobacteriota	Acidobacteriae	Bryobacterales	Bryobacteraceae		8	L1.bin.109
bagasse-all	Bacteria	Acidobacteriota	Acidobacteriae	UBA7540	UBA7540		5	L1.bin.50
bagasse-all	Bacteria	Acidobacteriota	Aminicenantia	Aminicenantales	RBG-16-66-30		3	L1.bin.18
bagasse-all	Bacteria	Actinobacteriota					1	L1.bin.49
bagasse-all	Bacteria	Actinobacteriota	RBG-13-55-18	RBG-13-55-18			1	L1.bin.31
plastics	Bacteria	Actinomycetota	Acidimicrobiia	Acidimicrobiales	JAAYBP01		8	PLS.PVC-P2_S42.bin.9
plastics	Bacteria	Actinomycetota	Acidimicrobiia	Acidimicrobiales	JAAYBP01	JAGQSI01	14	PLS.M.CASM.c.124
plastics	Bacteria	Actinomycetota	Acidimicrobiia	Acidimicrobiales	JAAYBP01	JAGQSI01	14	PLS.M.PVC_P7.s.2
plastics	Bacteria	Bacillota_E	Symbiobacteriia	Symbiobacteriales	Symbiobacteriaceae		1	PLS.PP-P10_S41.bin.15
plastics	Bacteria	Pseudomonadota	Alphaproteobacteria	Rhizobiales	Phreatobacteraceae	*Phreatobacter*	1	PLS.H.PVC_P11.m.16

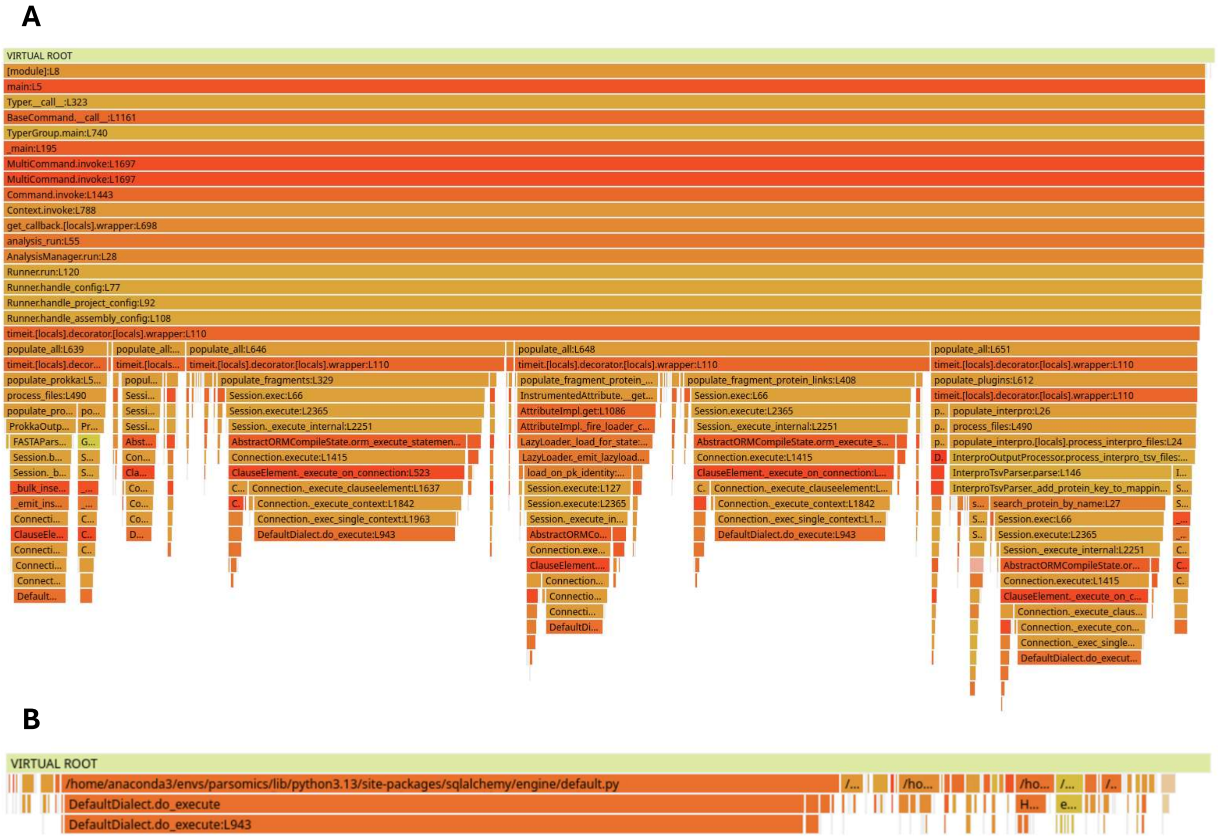

Figure S6: Benchmark results for *parsomics* generated using a metagenomic project containing 110 MAGs. (**A**) Top-down flamegraph showing execution time for each level of *parsomics-core* call stack, highlighting the relative duration of function calls to support performance analysis. (**B**) Flat tree visualization illustrating that the majority of execution time is spent on database interactions (through the *DefaultDialect.do_execute* function of the SQLAlchemy library). These results confirm that *parsomics* is an I/O bound application. These benchmarking profiles were generated using Austin (Tornetta 2024) and visualized with EasyView(Zhao, Chabbi and Liu 2023).

Table S3: Summary statistics for *parsomics* benchmark runs on six metagenomic projects varying in size, number of MAGs, and annotation complexity. These datasets demonstrate the framework's scalability and performance consistency across diverse workloads.

Project	**Project Description**	# Samples	# MAGs	Protein annotation tools	Data size (GB)	Time (h)	DB size (GB)
Bag10	Test dataset	1	10	4	0.14	0.08	0.14
Bag_full	Soil covered with sugarcane bagasse	1	111	2	0.80	0.42	0.86
PLS	Plastic-associated metagenome	24	186	2	3.70	1.16	3.94
CAMI I	CAMI High complexity	5	402	2	8.00	2.36	8.49
PBM	Marine Manatee microbiome	11	680	2	7.90	2.78	8.33
PBA_PBM	Amazonian and Marine Manatee microbiome	47	1215	3	12.10	5.11	11.00

Supplementary References

Davis M. SnakeViz: An in-browser Python profile viewer. 2024.

Roskind J, Mullender S, Rossum G. *The Python Profilers: CProfile*.

Tornetta G. Austin: Python frame stack sampler for CPython. 2024.

Zhao Q, Chabbi M, Liu X. EasyView: Bringing Performance Profiles into Integrated Development Environments. 2023.
